# Visual search for real‐world scenes in patients with Alzheimer's disease and amnestic mild cognitive impairment

**DOI:** 10.1002/brb3.3567

**Published:** 2024-06-06

**Authors:** Müge Akkoyun, Koray Koçoğlu, Hatice Eraslan Boz, Işıl Yağmur Tüfekci, Merve Ekin, Gülden Akdal

**Affiliations:** ^1^ Department of Neuroscience, Institute of Health Sciences Dokuz Eylul University Izmir Türkiye; ^2^ Department of Neurology, Unit of Neuropsychology Dokuz Eylul University Izmir Türkiye; ^3^ Department of Neurology, Faculty of Medicine Dokuz Eylul University Izmir Türkiye

**Keywords:** Alzheimer's disease, amnestic mild cognitive impairment, real‐world scenes, visual attention, visual search

## Abstract

**Background:**

Visual attention‐related processes that underlie visual search behavior are impaired in both the early stages of Alzheimer's disease (AD) and amnestic mild cognitive impairment (aMCI), which is considered a risk factor for AD. Although traditional computer‐based array tasks have been used to investigate visual search, information on the visual search patterns of AD and MCI patients in real‐world environments is limited.

**Aim:**

The objective of this study was to evaluate the differences in visual search behaviors among individuals with AD, aMCI, and healthy controls (HCs) in real‐world scenes.

**Materials and Methods:**

A total of 92 participants were enrolled, including 28 with AD, 32 with aMCI, and 32 HCs. During the visual search task, participants were instructed to look at a single target object amid distractors, and their eye movements were recorded.

**Results:**

The results indicate that patients with AD made more fixations on distractors and fewer fixations on the target, compared to patients with aMCI and HC groups. Additionally, AD patients had longer fixation durations on distractors and spent less time looking at the target than both patients with aMCI and HCs.

**Discussion:**

These findings suggest that visual search behavior is impaired in patients with AD and can be distinguished from aMCI and healthy individuals. For future studies, it is important to longitudinally monitor visual search behavior in the progression from aMCI to AD.

**Conclusion:**

Our study holds significance in elucidating the interplay between impairments in attention, visual processes, and other underlying cognitive processes, which contribute to the functional decline observed in individuals with AD and aMCI.

## 1 INTRODUCTION

Alzheimer's disease (AD) is a degenerative neurological disease that is typically characterized by early memory impairment, and studies also have reported deficits in other aspects of cognitive processes, such as attention, executive functions, and visuospatial abilities in the initial phase of the disease (McKhann et al., 2011; Perry & Hodges, 1999). Attention deficits in AD are a common cognitive problem except for memory impairment and affect maintaining independence in daily living activities (Perry & Hodges, 1999; Ramzaoui et al., 2018). Therefore, a comprehensive investigation of attentional deficits is essential to understand the process from healthy aging to AD and to monitor the progress (Mapstone et al., 2008). Mild cognitive impairment (MCI) consists of more than just the cognitive changes seen in normal aging. Especially, the amnestic MCI (aMCI) variant expressed by memory decline is a risk factor for AD (Petersen, 2003, 2004; Petersen et al., 2001). Thus, the focus of many studies has primarily been on the decline in memory in patients with aMCI. Nevertheless, alterations in non‐memory functions, such as attention deficit, may manifest in these individuals because of the risk of developing AD (McLaughlin et al., 2014).

Eye movements are an essential component of the visual process that provides information about how individuals perceive the environment (Klein & Ettinger, 2019). Moreover, measuring eye movement during goal‐directed behavior such as visual search can give valuable insights into potential disruptions in visual cognition in patient populations (Molitor et al., 2015). Visual search behavior is searching a target item among a set of distractor items within the visual scene (Wolfe, 1998a, 1998b). The efficacy of searching for a target item contains various complex capabilities, such as search strategy, ignoring distractors, speed of perception, and proficiency in shifting attention from one item to another (McLaughlin et al., 2010). Therefore, visual search tasks are widely used measures of visual attention ability (Chun & Wolfe, 2001).

Previous investigations on visual search in AD mostly utilized classical computer‐based tasks involving simple arrays, such as letters, numbers, or geometric shapes (Treisman, 1988; Wolfe, 1998a). Studies have shown that individuals with AD exhibit different patterns of visual search behavior, compared to healthy older adults. Specifically, AD patients tend to make fewer fixations toward the target and have longer fixation durations toward distractors, deficits in search accuracy, and make more errors for target detection during visual search tasks (Cormack et al., 2004; Foster et al., 1999; Greenwood et al., 1997; Moser et al., 1995; Nebes & Brady, 1989; Parasuraman et al., 1995, 2000; Pereira et al., 2020; Rösler et al., 2005, 2000; Tales et al., 2002, 2004; Tales, Haworth, et al., 2005). Despite the frequently used visual search paradigms in the literature exploring visual attention in AD patients and older adults, few studies have investigated the visual search capacity of individuals with MCI. Recently, studies indicate that patients with MCI experience a reduction in their ability to shift attention in visual search performance, specifically concerning top‐down attentional control (Perry & Hodges, 2003; Tales et al., 2005a). Patients with MCI were poorer at detecting the target when surrounded by distractors than healthy controls (HCs; Tales et al., 2011, 2005; Tales, Haworth, et al., 2005). In addition, AD and MCI patients had similar visual search patterns such as more fixation durations searching for the target and making more fixated on distractors (Pereira et al., 2020; Tales, Haworth, et al., 2005).

Searching for objects within real‐world scenes that portray everyday environments may give important information about visual search behavior in daily life for individuals with cognitive impairments such as AD (Land et al., 1999; Neargarder & Cronin‐Golomb, 2005; Ramzaoui et al., 2018). However, studies are quite limited in using real‐world scene tasks for evaluating visual search parameters in AD, and most of these studies did not use search tasks (Ramzaoui et al., 2018). Research findings showed difficulties in directing attention from the peripheral field to the target, more saccades toward distractors, displayed reduced accuracy, and spending more time engaging in target search behavior in AD (Boucart et al., 2014a, 2014b; Eraslan Boz et al., 2023; Lenoble et al., 2015; Neargarder & Cronin‐Golomb, 2005; Ramzaoui et al., 2022; Shakespeare et al., 2015; Vallejo et al., 2016). Studies evaluating the visual search behaviors of the aMCI group have found that, compared to healthy individuals, they tend to gaze at distractors for a longer duration and exhibit increased fixation (Eraslan Boz et al., 2023d; Pereira et al., 2020). However, due to methodological differences in these studies, there exists an insufficient body of studies as of yet to establish a consensus regarding visual search performance in individuals with aMCI. Therefore, our study aimed to investigate visual search patterns in real‐world settings in patients with AD and aMCI. Based on the observed alterations in visual attention in the early stages of the disease, we hypothesized that impaired visual search in real‐world scenes would be displayed in the aMCI and AD groups.

## 2 MATERIAL AND METHODS

### 2.1 Participants

A total of 92 participants, including 28 patients with AD dementia, 32 patients with aMCI, and 32 HCs were enrolled in the study. Participants were recruited from Dokuz Eylül University Hospital, Department of Neurology. Physical/neurological examinations, routine laboratory tests, and clinical history taking of the participant were performed by neurologists. Comprehensive neuropsychological tests for participants were fulfilled in the neuropsychology laboratory by neuropsychologists. Recording of eye movements was carried out in the Balance and Eye Movement Recording Laboratory of the Neurosciences Department. Patients with AD were included according to the National Institute of Neurological and Communicative Diseases and Stroke/Alzheimer's Disease and Related Disorders Association criteria for probable AD dementia in the study (McKhann et al., 2011). In addition, participants who scored 1 point on the Clinical Dementia Rating (CDR) scale (Gürvit & Baran, 2007; Hughes et al., 1982) and below 18 points on the Geriatric Depression Scale (GDS;(Ertan et al., 1997; Yesavage et al., 1982) and had no psychiatric or other neurological disease were included in the AD group.

The inclusion criteria for patients with aMCI in the study were: (1) complaint of cognitive decline informed by the patient and their relative, (2) had neuropsychological test scores below 1.5 standard deviations according to age and education norms (impairment in the memory domain or the impairment with other cognitive domains including memory domain), (3) preservation of activities of daily living, (4) these criteria do not fulfill the diagnostic criteria for dementia (Albert et al., 2011). Participants who scored 0.5 points on the CDR scale and below 18 points on the GDS and had no psychiatric or neurological disease were included in the aMCI group.

Healthy participants were selected according to the absence of psychiatric or neurological disease that may affect cognitive functions, below 18 points on the GDS, within age and educational cognitive test norms.

All participants with normal or corrected‐to‐normal vision and no hearing difficulties that might interfere with communication were included in the study to ensure cognitive testing and eye movement evaluations. Furthermore, participants with a history of stroke, head trauma, or cardiovascular diseases and taking any medication that could affect cognitive functions or eye movements were excluded from the study.

The study was conducted in accordance with the Declaration of Helsinki and approved by the Dokuz Eylül University Ethical Committee (Protocol Number: 2019/18‐32). Written informed consent was obtained from all participants.

### 2.2 Neuropsychological assessment

Comprehensive neuropsychological assessments were conducted to evaluate the cognitive functions of all participants. Verbal memory was assessed with the Verbal Memory Processes Test (Öktem, 1992), and the Rey Complex Figure Test (Meyers & Meyers, 1995; Ossterrieth, 1944; Rey, 1941; Varan et al., 2007) was used for visual memory. Attention was measured by Digit Span (Wechsler, 1987) and Trail Making Test (Cangöz et al., 2007; Reitan, 1958). Stroop Test (Karakaş, 2006; Stroop, 1935), Wisconsin Card Sorting Test (Berg, 1948; Heaton, 1981; Karakaş, 2006), Clock Drawing Test (Cesar et al., 2021; Dion et al., 2022; Rouleau et al., 1992), and phonemic and semantic fluency tests (Benton et al., 1978; Heaton et al., 1993; Martin et al., 1994; St‐Hilaire et al., 2016; Tumac, 1997) were used for executive function. Visuospatial functions were evaluated using the Judgement of Line Orientation test (Benton et al., 1978; Karakaş, 2006; Tranel et al., 2009) and the Benton Face Recognition Test (Benton & Allen, 1968; Keskinkılıç, 2008; Tranel et al., 2009). Language skills were assessed with the Boston Naming Test (Kaplan et al., 1983; Kurt et al., 2016; Morris et al., 1989). Mini‐Mental State Examination (MMSE) was used for evaluating global cognition (Folstein et al., 1975; Keskinoglu et al., 2009). The assessment of depressive symptoms in the participants was conducted using GDS (Ertan et al., 1997; Yesavage et al., 1982).

### 2.3 Eye movement recording

#### 2.3.1 Apparatus

EyeLink 1000 Plus eye‐tracker (SR Research, RRID: SCR_009602) was used for eye movement parameters recorded monocularly from the right eye with high saccade sensitivity and a sampling rate of 1000 Hz. Paradigms were shown on the Phillips 226E9Q screen [width: 290 (1900.25 pixels) × 170 (1048.09 pixels)] with a refresh rate is 60 Hz., and the response time of the screen is 4 ms, on which the recordings are made. The screen resolution is 1920 × 1080 pixels, and the aspect ratio is 16:9. Eye movement recordings were performed in a quiet and dimly lit room. The participant is seated at a distance of 85 cm from the screen. Participants were positioned on a chin rest with head support. Nine‐point calibration and validation procedures were used for all participants before the visual search task. These procedures were used to adjust the measurement system to ensure accurate tracking of the eye movements. Participants were given instructions to refrain from moving their heads or closing their eyes.

#### 2.3.2 Visual search task and procedure

Real‐world scene stimuli were composed of three trials of kitchen pictures. Photographs were taken with the CANON EOS M50 by the research team of Dokuz Eylül University Institute of Health Sciences, Department of Neurosciences, Balance and Eye Movement Recording Laboratory.

**FIGURE 1:**
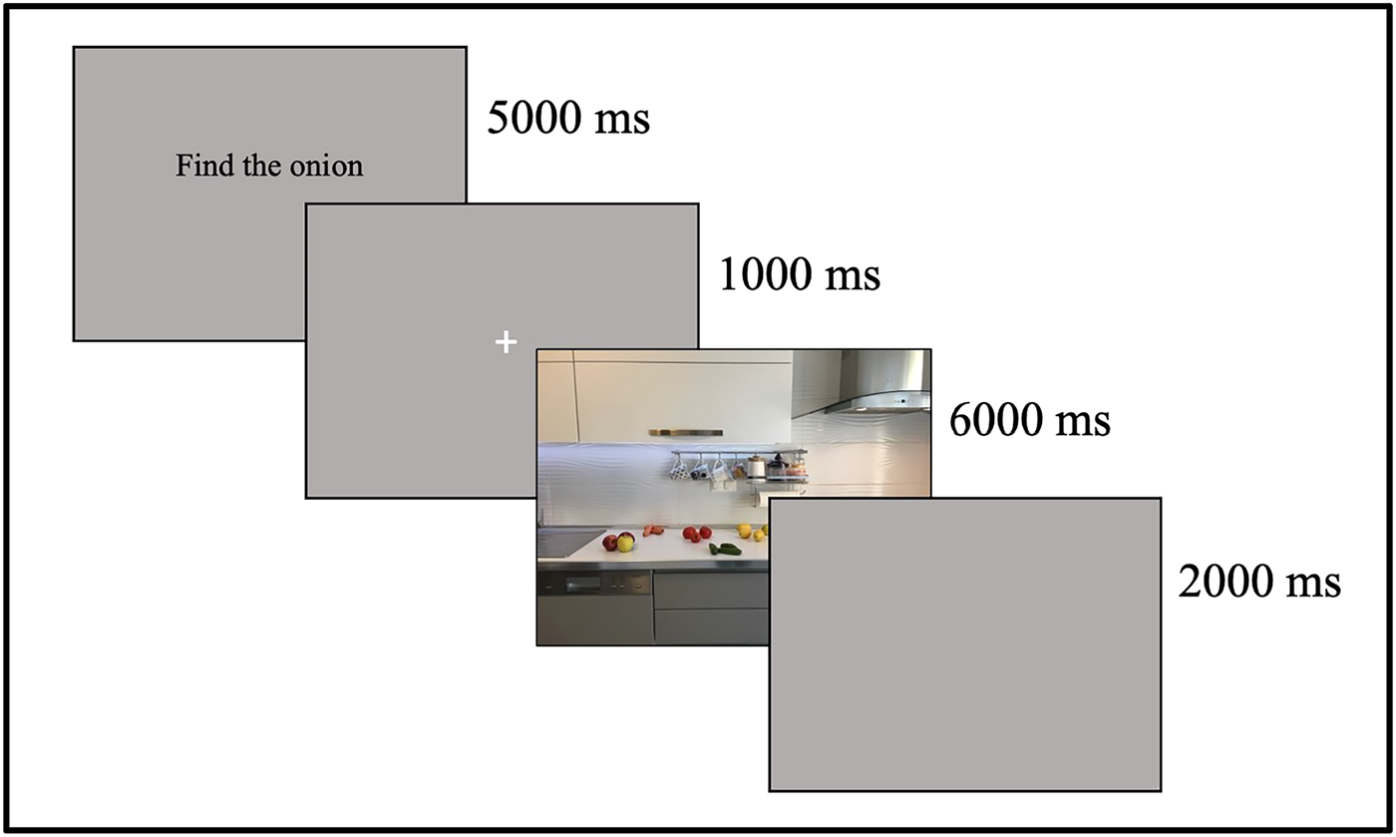
Representation of the visual search tasks.

**FIGURE 2:**
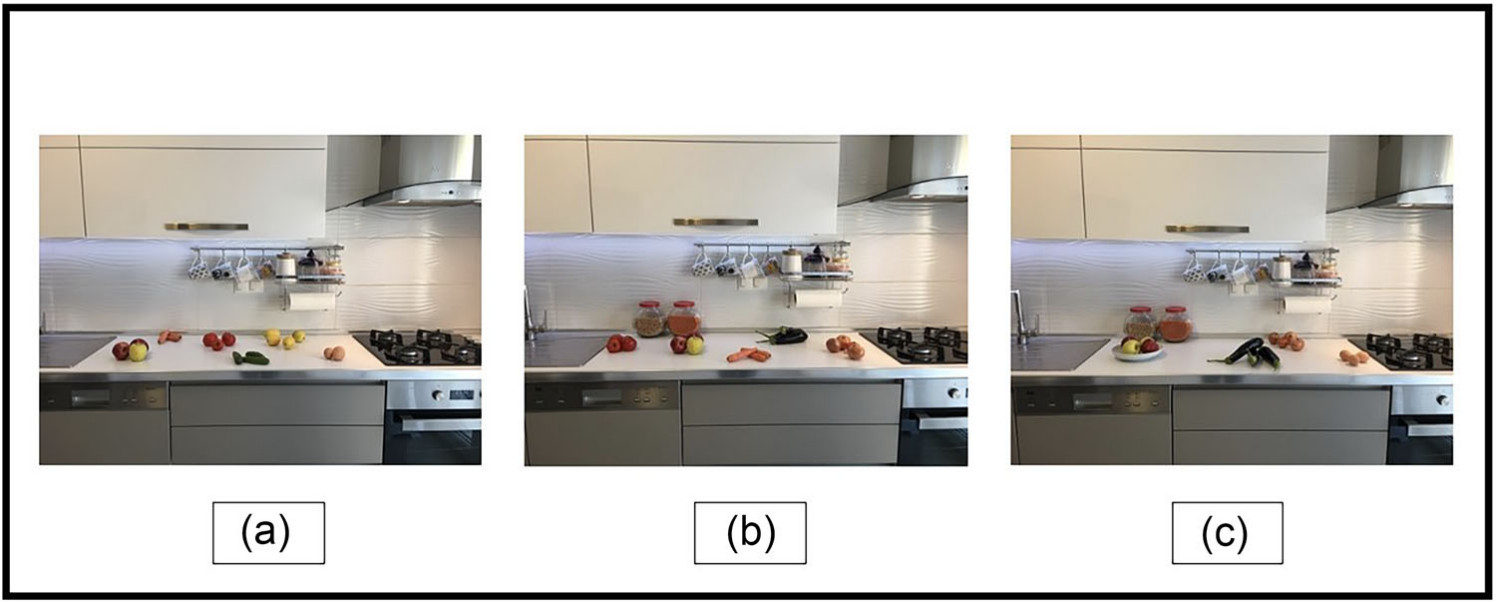
Images of the visual search task used for the study. The real‐world scenes task to find a single vegetable in kitchen scenes. (a) Look at the onion, (b) look at the tomato, and (c) look at the eggplant.

During the visual search paradigm, participants were instructed to look at a specific object (eggplant, onion, and tomato) in visual scenes. Each visual scene was shown for 6000 ms. A gray (Red‐Green‐Blue Color Model, *RGB*,127, 127, 127, 255) screen was shown for 2000 ms between trials. Afterward, the instructions for the materials to be found in the pictures were shown on the screen for 5000 ms. The instruction was presented both visually and verbally. The instruction screen was presented in black text on a gray (RGB 127, 127, 127, 255) background. Then a white fixation cross was displayed on a gray background for 1000 ms (Figure 1). The pictures and instructions shown in the task are presented in Figure 2. The experimental tasks were designed and programmed using the Experiment Builder Software (SR Research).

#### 2.3.3 Data analysis

Extracted and analysis of recording eye movement raw data was performed with the EyeLink Data Viewer software package (SR Research, version 3.2.1). The number of fixations and fixation duration parameters were analyzed in this experiment: These parameters were measured separately for two types of regions of interest (ROI): target‐located ROI and distractors‐located ROI. Fixation durations were measured as the total time of fixation for each ROI. The number of fixations represented the sum of the fixation counts for each ROI. Heat maps were created to explore visual search behavior during the viewing of real‐world scenes. Each heat map was generated from the average fixation duration of all groups with Data Viewer. Blinks and artifacts as outliers were excluded from the analysis.

### 2.4 Statistical analysis

The Statistical Package for Social Sciences (SPSS 25.0) program was used to analyze all study data. The normality distribution of eye movement parameters, cognitive domains, and demographic characteristics was analyzed by using Kurtosis and Skewness Coefficients (*z*‐score < 1.96) and the Kolmogorov–Smirnov. The Chi‐square test was used for analyzing group differences in sex. Independent samples *t*‐test was used for disease duration of aMCI and AD patients.

Demographic variables, MMSE scores, and composite *z*‐score means of cognitive domains patients were analyzed with one‐way analysis of variance (one‐way ANOVA). Pairwise comparisons of cognitive domains were analyzed with Bonferroni correction. The effect size of pairwise comparisons was measured by Cohen's *“d”* (Cohen, 1988). Repeated measures ANOVA was conducted on eye movement parameters. The ANOVA model had one between‐subjects factor (group; AD, aMCI, HC) and one within‐subjects factor (stimulus; target, distractor). Pairwise comparisons with Bonferroni were used for within‐subjects, between‐subjects, and interaction results. Significance was accepted at *p* < .05 for all statistical analyses.

## 3 RESULTS

### 3.1 Demographic features and neuropsychological assessment

There were no significant differences in age, sex, or education between groups; GDS scores and the duration of the disease showed no significant difference between patients with AD and aMCI. A comprehensive neuropsychological test battery was performed to evaluate five cognitive domains, and the results were converted into a composite *z*‐score. In addition, global cognition was measured with the MMSE. There were significant differences in the neuropsychological test results between all groups (Table 1).

**TABLE 1:** Demographic features and neuropsychological assessment.

	HC	aMCI	AD	*p‐values*	Pairwise	Cohen's *d*
	(*n* = 32)	(*n* = 32)	(*n* = 28)		Comparisons	
*Demographic*						
Age (year)	68.59 ± 6.24	70.69 ± 6.91	72.75 ± 7.95	.079^a^	‐	‐
Sex (female/male)	21/11	18/15	15/15	.326^b^	‐	‐
Education (year)	11.69 ± 2.95	9.69 ± 4.92	9.36 ± 4.76	.074^a^	‐	‐
Duration of disease (month)	‐	27.44 ± 22.69	31.25 ± 22.58	.518^c^	‐	‐
GDS	7.63 ± 5.16	8.88 ± 5.31	5.85 ± 3.88	.069^a^	‐	‐
*General Cognitive Status*						
MMSE	29.16 ± 0.98	26.56 ± 1.19	21.64 ± 2.64	<.001	aMCI‐HC: *p* <.001	−2.385
					AD‐HC: *p* <.001	−3.879
					aMCI‐AD: *p* <.001	−2.459
*Cognitive Domains*						
Memory	0.67 ± 0.27	−0.07 ± 0.27	−0.63 ± 0.28	<.001	aMCI‐HC: *p* <.001	−2.740
					AD‐HC: *p* <.001	−4.732
					aMCI‐AD: *p* <.001	−2.038
Attention	0.59 ± 0.38	−0.13 ± 0.47	−0.48 ± 0.48	<.001	aMCI‐HC: *p* <.001	−1.684
					AD‐HC: *p* <.001	−2.491
					aMCI‐AD: *p* =.007	−0.737
Executive functions	0.34 ± 0.17	0.03 ± 0.28	−0.40 ± 0.41	<.001	aMCI‐HC: *p* <.001	−1.338
					AD‐HC: *p* <.001	−2.417
					aMCI‐AD: *p* <.001	−1.240
Visuospatial functions	0.63 ± 0.33	−0.03 ± 0.59	−0.64 ± 0.66	<.001	aMCI‐HC: *p* =.001	−1.380
					AD‐HC: *p* <.001	−2.486
					aMCI‐AD: *p* =.004	−0.978
Language	0.30 ± 0.50	−0.06 ± 0.42	−0.25 ± 0.63	<.001	aMCI‐HC: *p* =.018	−0.779
					AD‐HC: *p* <.001	−0.974
					aMCI‐AD: *p* =.522	‐

*Note*: The values are presented mean ± standard deviation. Statistical differences are shown in bold, *p *<.05.

^a^ One‐way analysis of variance (ANOVA).

^b^ Chi‐quare Test.

^c^ Independent samples t‐test.

^d^One‐way ANOVA post‐hoc with Bonferroni correction, Cohen's *d*: effect size.

Abbreviations: AD, Alzheimer's disease; aMCI, amnestic mild cognitive impairment; GDS, Geriatric Depression Scale; HC, Healthy control; MMSE, Mini‐Mental State Examination.

### 3.2 Visual search results

#### 3.2.1 The number of fixations

**FIGURE 3:**
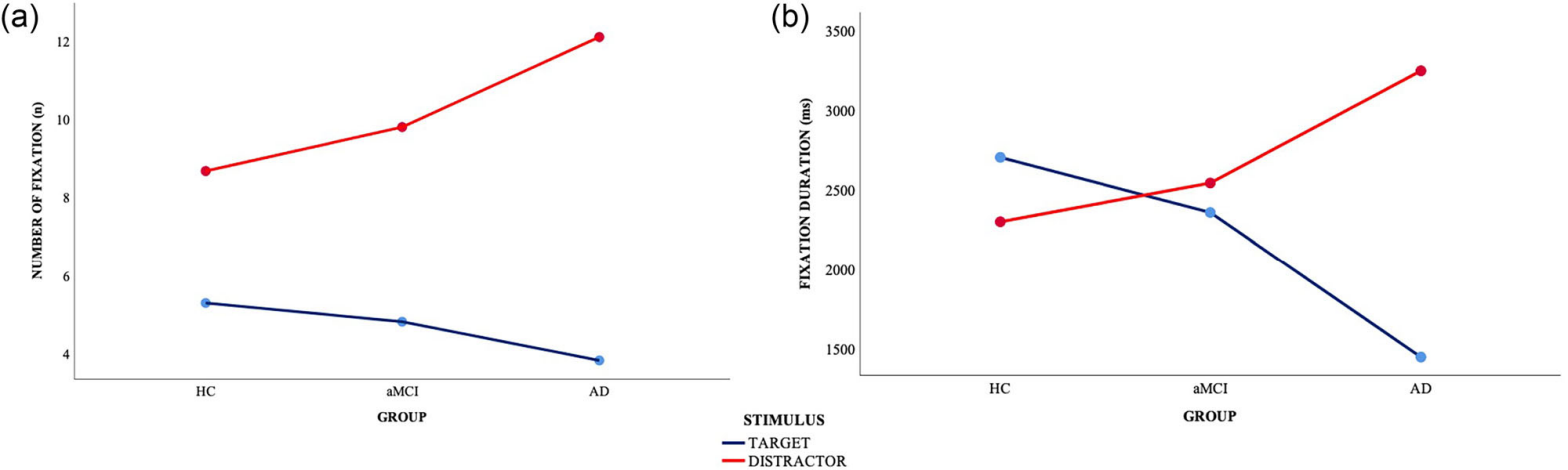
Eye movement parameters in HCs, aMCI, and AD patients: (a) number of fixations in the visual search task and (b) fixation duration in the visual search task. AD, Alzheimer's disease; aMCI, amnestic mild cognitive impairment; HC, healthy control.

A main effect of “Stimulus” was found on the number of fixations [*F* (1, 89) = 106.903, *p* < .001, *η_p_* ^2^ = 0.55], and also, there was a marginally significant main effect of “Group” [*F* (1, 89) = 3.079, *p* = .051, *η_p_* ^2 ^= 0.06]. Participants were fewer looking at the target than distractors (*p* < .001). There was a statistically significant “Stimulus × Group” interaction [*F* (1, 89) = 6.966, *p* = .002, *η_p_* ^2^ = 0.14]. HCs were slightly significantly more looking at the target than the AD group (*p* = .050). Both patients with aMCI and HC groups were significantly less looking at the distractors than the AD group (respectively, *p* = .041; *p* < .001; Table 2, Figure 3).

**TABLE 2:** Eye movement results of participants in the visual search task.

	HC (*N* = 32)	aMCI (*N* = 32)	AD (*N* = 28)	*p‐*values	Group	Stimulus	Interaction	Pairwise comparisons
Number of fixation (*n*)	Target	5.29 ± 2.70	4.81 ± 2.05	3.82 ± 2.15	.051	<.001	.002	HC‐AD: 0.050*
	Distractor	8.67 ± 3.38	9.80 ± 3.92	12.10 ± 3.35				HC‐AD: 0.001**
								aMCI‐AD: 0.041**
Fixation duration (*ms*)	Target	2704.66 ± 1236.42	2358.85 ± 1229.73	1450.17 ± 949.26	<.001	.025	.001	HC‐AD: < 0.001*
								aMCI‐AD: 0.009*
	Distractor	2300.70 ± 1210.97	2543.79 ± 1096.34	3247.34 ± 840.77				HC‐AD: 0.003**
								aMCI‐AD: 0.038**

*Note*: Values are shown as mean ± standard deviation. Statistical differences are shown in bold, *p *<.05.

Repeated measures ANOVA controlled for between‐subject effect are used in all parameters.

Bonferroni correction used for pairwise comparisons are presented for statistically significant interaction effects.

Abbreviations: AD, Alzheimer's disease; aMCI, amnestic mild cognitive impairment; HC, healthy control; *n*, number; ms, milliseconds.

*Target; **Distractor.

#### 3.2.2 Fixation duration

A significant main effect of “Group” [*F* (1, 89) = 8.490, *p* < .001, *η_p_^2^* = 0.16] and “Stimulus” [*F* (1, 89) = 5.210, *p* = .025, *η_p_^2^* = 0.055] were found for the fixation durations. Both HC and aMCI groups spent more time fixating on the ROI than the AD group (respectively, *p* < .001; *p* = .024), and participants spent more time fixating on the distractors than the target (*p* = .025). Furthermore, there was a “Stimulus × Group” interaction effect for the fixation duration [*F* (1, 89) = 7.844, *p* = .001, *η_p_^2^* = 0.15]. The pairwise comparisons revealed that the AD group spent less time fixating on the target than both HC (*p* < .001) and aMCI groups (*p* = .009), and they significantly spent more time looking at the distractors compared to the aMCI patients and HCs (respectively, *p* < .038; *p* = .003; Table 2, Figure 3)

**FIGURE 4:**
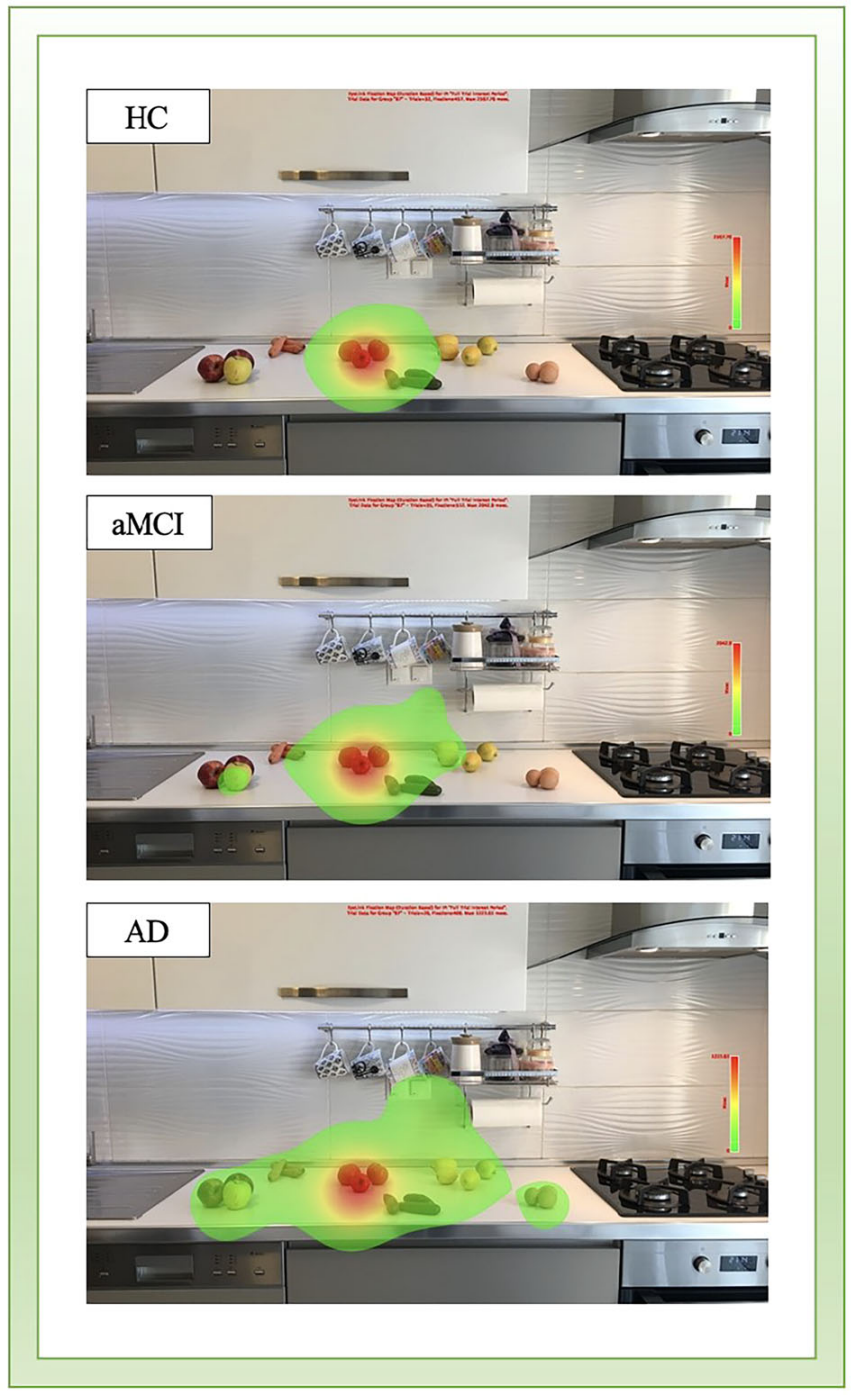
Representative heatmap of fixations in the visual search task for each group. Participants were asked to find and look at the tomato in the kitchen scene. The red areas represent the places where the participants looked most intensely, depending on the fixation duration. The green areas represent the searching areas with a less fixation duration. AD, Alzheimer's disease; aMCI, amnestic mild cognitive impairment; HC, healthy control.

Each fixation of the participants was used to construct a heat map. The heat map presented different color schemes indicating the fixation duration made in any ROI. Across all groups, fixations were most intensely on the target‐located ROI, whereas the pattern of fixations was different between groups. Patients with AD and aMCI more concentrated on the distractors than HCs, while searching for the target. Differences in the heat map between all groups are shown in Figure 4.

## 4 DISCUSSION

The present study aimed to examine visual search behavior using real‐world scenes in AD and aMCI. Our findings indicated that the AD group had fixated on the distractors more than the aMCI and HC groups. In addition, the results showed that HCs and aMCI patients spent more time looking at the target, while AD patients spent more time looking at the distractors during the search.

In natural settings, the visual environment tends to be cluttered with distractors, making the process of locating and processing an object within a visual scene a demanding task. This often requires attention shifting throughout the visual scene, examining numerous items until the target is found. The presence of higher the number of distractors in the scene, the more time it takes to find the target (Chun & Wolfe, 2001; Tales & Porter, 2008; Tales, Haworth, et al., 2005). In our study, we found that participants showed increased fixation duration and made a higher number of fixations to the distractors than the target. AD group looked at distractors longer time while searching for the target than both the aMCI and HC groups. Therefore, patients with AD spent less time fixating on the target. Previous studies have also found that the duration of looking at distractors in the AD group significantly increased while seeking the target, compared to HCs, in line with our results (Eraslan Boz et al., 2023d; Ishızaki et al., 2013; Neargarder et al., 2005; Parasuraman et al., 1992; Ramzaoui et al., 2022; Rösler et al., 2005). These studies stated that, while this may be linked to difficulties in shifting attention between items, the increased fixation time during searching may also be a result of long‐time target processing. Accurate perception of objects within a complex visual environment is dependent on the engagement of spatial attention. Attention deficit can lead to impairments in target processing (Boucart et al., 2014a; Pereira et al., 2020). The current study found that the AD group exhibited more fixations on distractors than the aMCI and HC groups and fixated less on the target than the HCs. Similarly, previous studies indicated an increased number of fixations to distractors affecting visual search efficiency in patients with AD (Cormack et al., 2004; Eraslan Boz et al., 2023d; Pereira et al., 2020; Ramzaoui et al., 2022; Tales et al., 2002).

Research on patients with MCI has highlighted the presence of impairment in top‐down attentional control (Perry & Hodges, 2003), focused and divided attention deficit (Levinoff et al., 2005; Okonkwo et al., 2008), disengagement of attention, and visual search deficiency (Pereira et al., 2020; Tales, Haworth, et al., 2005; Tales, Snowden, et al., 2005). Specifically, individuals with aMCI characterized by memory impairment have shown impairment in visual attention‐related processes and significant reductions in visual attention performance, compared to cognitively healthy individuals (Okonkwo et al., 2008; Perry & Hodges, 2003). In a visual search study, MCI and AD patients showed similar visual search patterns. AD and MCI patients looked at distractors longer than the target stimulus and made more fixations, compared to HCs (Pereira et al., 2020). Tales, Haworth, et al. (2005) found increased reaction time (RT) in MCI than HCs and less RT, compared to AD patients when the target was surrounded by the distractors. Studies have indicated that these results may be due to impairments in the processing of visual information in MCI and may display the presence of a visual search effect indicating a functional impairment other than memory (Pereira et al., 2020; Tales et al., 2005a; 2011). In the visual search study by Eraslan Boz et al. (2023d), which is the most related study to our research, participants were asked to find the target among stimuli consisting of fragments of nine photographs taken from natural scenes. Results of the study showed AD and aMCI patients looked more and longer distractors, compared to HCs. Both patient groups demonstrated a decline in visual search performance. On the contrary, we found that the visual search measures of patients with MCI were similar to HCs. The MCI group looked at the distractors for less time and made fewer fixations, and also had a higher number of fixations to the target stimulus and longer fixation durations than the AD group. However, the MCI group did not significantly differ from the AD and HC groups in terms of visual scanning behavior, except for the total fixation duration. The MCI group performed at an intermediate level between the two groups. Studies on MCI patients have used visual search tasks involving encoding and recognition phases or discrimination tasks in which distractors have the same form as the target or have used visual scanning tasks in natural environments (Eraslan Boz et al., 2023c; Pereira et al., 2020; Tales et al., 2011; Tales, Haworth, et al., 2005). In our study, we evaluated visual search behavior in real‐world scenes in AD, aMCI, and HCs. The absence of significant differences between the visual search patterns observed in the MCI and HC groups may be attributed to differences in the methodologies used.

In summary, we have shown visual search alterations using scenes representing the real world in AD and aMCI. Therefore, our study may offer insight into future studies investigating how cognitive processes affect visual search in natural environments in AD and aMCI and investigating changes in the progression of these patient groups. The relatively small number of trials of the visual scanning paradigm can be considered a limitation of our study. In future studies, by increasing the number of trials, visual scanning performances in more complex real‐world scenes containing more than one target stimulus can be evaluated.

The use of eye‐tracking technology to evaluate visual search behavior can be a valuable tool in characterizing oculomotor behavior and visual attention in patients with MCI and AD. Utilizing real‐world scenes can improve the ecological validity of visual search tasks, leading to a better generalization of findings to real‐world settings.

## AUTHOR CONTRIBUTIONS

**Müge Akkoyun**: Conceptualization; investigation; formal analysis; writing—original draft; writing—review and editing. **Koray Koçoğlu**: Conceptualization; methodology; data curation; formal analysis; writing—review and editing. **Hatice Eraslan Boz**: Methodology; investigation; writing—review and editing. **Işıl Yağmur Tüfekci**: Conceptualization; visualization; investigation. **Merve Ekin**: Conceptualization; writing—review and editing; investigation. **Gülden Akdal**: Conceptualization; Supervision; writing—original draft; writing—review and editing.

## CONFLICT OF INTEREST STATEMENT

The authors declare no conflicts of interest.

### PEER REVIEW

The peer review history for this article is available at https://publons.com/publon/10.1002/brb3.3567.

## Data Availability

The data that support the findings of this study are available from the corresponding author upon reasonable request.
